# Predicting incidence of hepatitis E using machine learning in Jiangsu Province, China

**DOI:** 10.1017/S0950268822001303

**Published:** 2022-07-28

**Authors:** Xiaoqing Cheng, Wendong Liu, Xuefeng Zhang, Minghao Wang, Changjun Bao, Tianxing Wu

**Affiliations:** 1 Jiangsu Provincial Centre for Disease Control and Prevention (Jiangsu Institution of Public health), Nanjing, Jiangsu, China; 2 Chinese Field Epidemiology Training Program, Chinese Center for Disease Control and Prevention, Beijing, China; 3 School of Computer Science and Engineering, Southeast University, Nanjing, China

**Keywords:** Forecast, hepatitis E, mathematical model

## Abstract

Hepatitis E is an increasingly serious worldwide public health problem that has attracted extensive attention. It is necessary to accurately predict the incidence of hepatitis E to better plan ahead for future medical care. In this study, we developed a Bi-LSTM model that incorporated meteorological factors to predict the prevalence of hepatitis E. The hepatitis E data used in this study are collected from January 2005 to March 2017 by Jiangsu Provincial Center for Disease Control and Prevention. ARIMA, GBDT, SVM, LSTM and Bi-LSTM models are adopted in this study. The data from January 2009 to September 2014 are used as the training set to fit models, and data from October 2014 to March 2017 are used as the testing set to evaluate the predicting accuracy of different models. Selecting models and evaluating the effectiveness of the models are based on mean absolute per cent error (MAPE), root mean square error (RMSE) and mean absolute error (MAE). A total of 44 923 cases of hepatitis E are detected in Jiangsu Province from January 2005 to March 2017. The average monthly incidence rate is 0.35 per 100 000 persons in Jiangsu Province. Incorporating meteorological factors of temperature, water vapour pressure, and rainfall as a combination into the Bi-LSTM Model achieved the state-of-the-art performance in predicting the monthly incidence of hepatitis E, in which RMSE is 0.044, MAPE is 11.88%, and MAE is 0.0377. The Bi-LSTM model with the meteorological factors of temperature, water vapour pressure, and rainfall can fully extract the linear and non-linear information in the hepatitis E incidence data, and has significantly improved the interpretability, learning ability, generalisability and prediction accuracy.

Hepatitis E is a new zoonotic disease caused by the hepatitis E virus (HEV). The clinical manifestations of hepatitis E are similar to those of hepatitis A, such as fatigue, anorexia, and jaundice, but the severity of symptoms and mortality of hepatitis E are higher than those of hepatitis A [1]. Humans are generally susceptible to HEV, but the virus mainly infects people aged 15–60 years. The mortality rate of the general population is from 1% to 3%, and the mortality rate of pregnant patients is from 5% to 25%. It can also cause neonatal hepatitis E or even death by vertical transmission [2].

Hepatitis E infection has a global distribution, but mainly in India, China, Pakistan, Mexico, and some other countries in Asia and Africa [3]. In the past decade, the occasional outbreaks of hepatitis E are on the rise in some high-income countries. A growing number of local sporadic cases of hepatitis E that the route of infection cannot be determined are threatening human health [4]. According to estimates from the study on the global burden of hepatitis E, there are approximately 20 million hepatitis E infections each year, resulting in more than 3 million symptomatic hepatitis E cases, and 55 000 hepatitis E-related deaths, which makes it an important public health concern [5].

At present, there are mainly four genotypes of HEV. Genotypes 1 and 2 cause interpersonal outbreaks or epidemics, whereas genotypes 3 and 4 mainly infect several species of mammals (e.g. pigs, sheep, etc.), but at the same time, they also infect humans under certain conditions, causing sporadic hepatitis E [6, 7]. The hepatitis E virus is mainly transmitted by contaminated water and food through the fecal-oral route and it is verified that meteorological factors are related to the incidence of hepatitis E, as climate change will influence the environment, which may affect the quality of water and food [8–10].

In China, the areas with the highest incidence of hepatitis E are mainly in the northwest and the east, and Jiangsu Province is one of the areas [11]. Therefore, predicting the incidence of hepatitis E is quite indispensable. However, the existing disease surveillance information management system lacks an effective prediction and early warning mechanism. The establishment of a scientific, dependable, robust mathematical model can effectively solve this problem.

Currently, most researchers utilised the autoregressive integrated moving average (ARIMA) model to predict the incidence of hepatitis E [12, 13]. However, the result might be unsatisfactory due to data linearity requirements. To tackle this problem, non-linear machine learning models, including support vector machine (SVM) [14], gradient boosting decision tree (GBDT) [15], back-propagation neural networks (BPNN) [16] and long short-term memory (LSTM) [14], are adopted to the prediction and early warning of hepatitis E. At present, the state-of-the-art model is LSTM used by Guo *et al*. [14]. This model can not only accurately capture the features of sequential data, but also effectively avoid the problems of vanishing gradients and exploding gradients on traditional recurrent neural networks. However, Guo *et al*. only use the past monthly incidence of hepatitis E to predict the incidence for the next month, and it cannot correct the current prediction with the input of the next time point. Besides, meteorological factors are not considered in their model [15]. These cause that the proposed LSTM model has much room for improvement.

Therefore, taking the above-mentioned problems, we propose a new Bi-LSTM model with various meteorological factors in this paper to predict the incidence of hepatitis E. Meanwhile, we compare our proposed model with existing models using ARIMA, SVM, LSTM and GBDT, aiming to provide the scientific basis for more effective hepatitis E incidence prediction, which also facilitates the development of the early warning system and the prevention strategies for hepatitis E in Jiangsu Province.

## Method

### Data source

The hepatitis E data used in this paper are collected from January 2005 to March 2017 by Jiangsu Provincial Center for Disease Control and Prevention. This dataset records the monthly incidence of hepatitis E in Jiangsu Province (P. R. China). Annual data of the demographic are obtained from Jiangsu Statistical Yearbook. The meteorological dataset is abstracted from the Jiangsu Meteorological Service Center, which contains the statistical data of 24 meteorological stations. We take the average value of the meteorological data observed by each station as the predicting value of monthly meteorological data.

### The Bi-LSTM model

#### Model structure

**Fig. 1.**
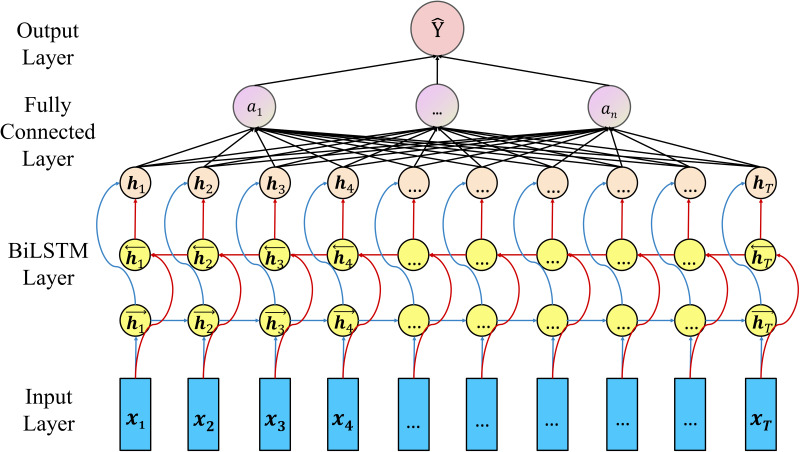
The structure of our proposed model.

As shown in Figure 1, four layers are constructed in the Bi-LSTM model, which are the Input Layer, Bi-LSTM Layer, Fully Connected Layer and Output Layer, respectively. The input of the model includes monthly feature vectors in the past, each of which is composed of monthly incidence of hepatitis E, monthly average temperature, monthly average water vapour pressure, etc. Such sequential vectors are entered into the Bi-LSTM Layer, which can optimise the prediction from both the previous input and the following data. This characteristic makes our model more robust and transferable. After the vector output by Bi-LSTM passes through the fully connected layer, the output result of the hepatitis E incidence rate of the current month can be obtained.

#### Model prediction

After preprocessing, we take the monthly incidence of hepatitis E and meteorological factors as monthly feature vectors. For each vector ***x***_*t*_, we use a Min-Max-Scaler to normalise all dimensions. After setting the timestep *T*, we use previous *T* months' feature vectors to predict the incidence of hepatitis E for the current month. The Bi-LSTM Layer includes two steps. The input sequence is entered into the LSTM cells in the forward step, and after that, the reverse form of the input sequence is fed to other LSTM cells, which is called the backward step. 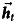 and 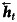 are used to represent the output in each step. The output of the Bi-LSTM layer is denoted as ***h***_*t*_.

**Fig. 2.**
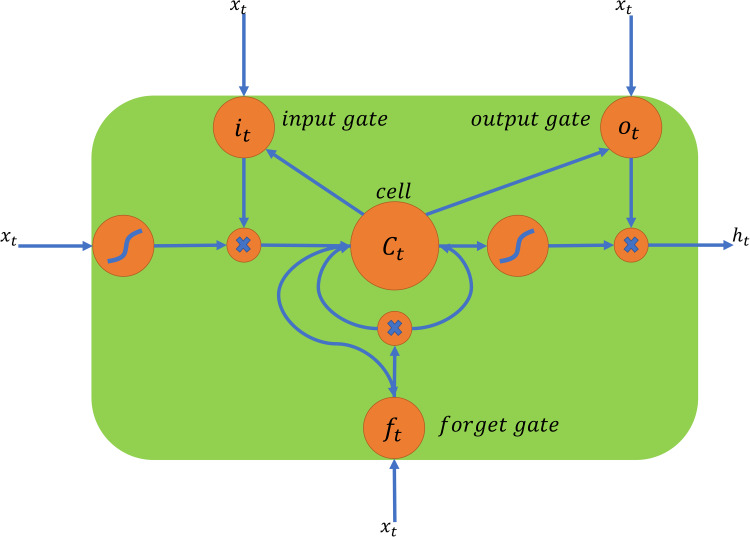
The structure of a LSTM cell.

During the forward step, the input sequence is fed to the LSTM cells, each of which consists of three gates, as shown in Figure 2. The input gate generates a value ***i***_*t*_ between 0 and 1 to determine how much new information needs to be retained. The forget gate generates a value ***f***_*t*_ between 0 and 1 to decide how much information should be neglected from the previous memory. With current input ***x***_*t*_ ∈ ℝ^*N*×1^ and previous state 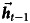, we get the candidate for new information 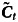 and the new state ***C***_*t*_, where *N* is the size of features. The output gate generates a value ***o***_*t*_ between 0 and 1 to determine how much information in the cell state will make sense, and finally gets the output information 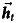 of the cell. The inherent logic of a LSTM cell is described by the following six equations.1
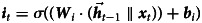
2
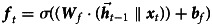
3
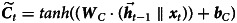
4
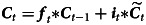
5
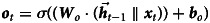
6
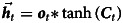
***W***_*i*_, ***W***_*f*_, ***W***_*C*_, ***W***_*o*_ ∈ ℝ^*u*×2*N*^ represent the weight matrices, *u* is the hidden size of the LSTM layer, ***b***_*i*_, ***b***_*f*_, ***b***_*C*_, ***b***_*o*_ ∈ ℝ^*u*^ represent the bias vectors, 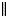 means vector concatenation, *σ* is the sigmoid function, and *tanh* is the hyperbolic tangent function. Note that the backward equation can be derived similarly by replacing 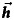 with 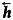.

After these two steps, the result of the Bi-LSTM layer is calculated by the following equation:7
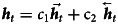
***h***_*t*_ represents the output of the Bi-LSTM layer, *c*_1_ and *c*_2_ are weights of two steps respectively.

For the Fully Connected Layer, we have:8
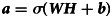
where ***a*** is the output vector, 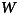 is the weight matrix between the Bi-LSTM layer and the MLP Layer, ***H*** is the concatenation of ***h***_1_, …, ***h***_*T*_, ***b*** is the bias vector, and *σ* represents the activation function, which the sigmoid function is used in this layer. All of the neurons are fed into the Output Layer and this Layer sums all of the information by this equation:9
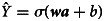
where 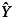 is the final result, ***a*** is the output of the Fully Connected Layer and ***w*** is the weight vector between the Fully Connected Layer and the Output Layer, *b* is the bias value, and *σ* represents the activation function which is also the sigmoid function.

### Model evaluation

The ARIMA, GBDT, SVM, LSTM, and the Bi-LSTM models are adopted in this study. The data from January 2009 to September 2014 are used as the training set to fit models, and data from October 2014 to March 2017 are used as the testing set to evaluate the prediction accuracy of different models. We use three standards, mean absolute per cent error (MAPE), root mean square error (RMSE) and mean absolute error (MAE), to estimate the results, compare the performance of these three models, and evaluate the influence of each meteorological factor. RMSE represents the sample standard deviation of the difference between the predicted value and the observed value. When the predicted value is completely consistent with the true value, i.e., RMSE is equal to 0, it is a perfect model. MAPE and MAE are also used to evaluate the model, and the less the value, the more accurate the model. The formula of each value is shown below.10
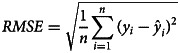
11
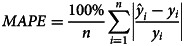
12
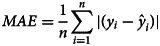
where *n* represents the number of months, *y*_*i*_ and 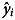 are the true incidence and the observed incidence of the *i*-th month, respectively.

### Statistical software

All statistical analyses are performed using Python software version 3.5.0. The ARIMA model is built using the pmdarima library, and the LSTM and the BiLSTM model are built using the tensorflow library.

## Results

### General description

**Fig. 3.**
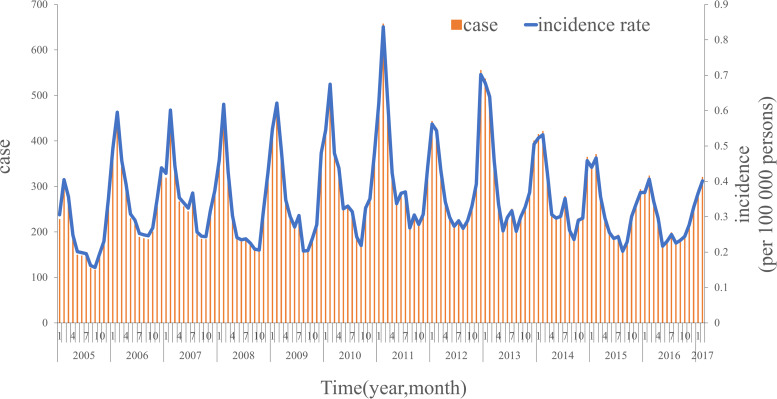
The incidence of hepatitis E in Jiangsu Province from 01.2005 to 03.2017.

A total of 44 923 cases of hepatitis E are detected in Jiangsu Province, from January 2005 to March 2017. The average monthly incidence rate is 0.35 per 100 000 persons in Jiangsu Province, as shown in Figure 3. The monthly incidence rate of hepatitis E varied seasonally, peaking in January through March.

### Model fitting

#### Bi-LSTM model

For the hyperparameter setting, the timestep is set to 2. The test scale is set to 30 months. The hidden neuron is set to 6. The epoch is set to 128. The batch size is set to 32. The optimizer is set to Adam, and the loss function is set to CrossEntropy. These are the optimal Hyper-parameters when using the monthly incidence of hepatitis E from January 2005 to September 2014 as the training set in the Bi-LSTM model. The models ARIMA, GBDT, SVM, LSTM and Bi-LSTM are employed to predict the monthly incidence of hepatitis E from October 2014 to March 2017. The comparison of three metrics of the models is shown in Table 1.

**Table 1.** Results of five models for monthly incidence of hepatitis E prediction

	RMSE	MAPE (%)	MAE
ARIMA	0.139	48.23	0.130
SVM	0.074	19.46	0.061
GBDT	0.097	23.32	0.078
LSTM	0.060	19.27	0.052
Bi-LSTM	0.054	15.78	0.044

#### Bi-LSTM model + meteorological factors

**Fig. 4.**
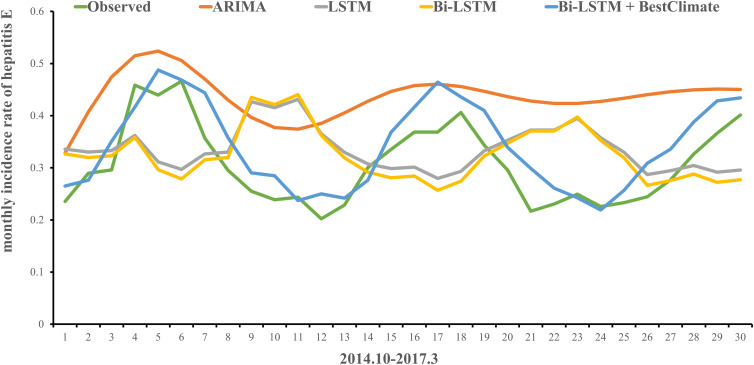
Plot of observed monthly incidence of hepatitis E and predicted values via different models.

Meteorological factors of temperature, atmosphere, water vapour pressure, rainfall, wind speed and humidity are included in the Bi-LSTM Model. Meteorological factors of *temperature* *+* *water vapour Pressure* *+* *rainfall* as a combination in the Bi-LSTM Model is the optimal among the 63 combinations. Table 2 shows the top 15 combinations. The comparison of three metrics of the models is shown in Table 3 and Figure 4, demonstrating the observed incidence curve and predicting curves of the models.

**Table 2.** Combinations of meteorological factors, ascending by RMSE

combinations of meteorological factors	RMSE
Temperature + Water Vapour Pressure + Rainfall	0.044
Water Vapour Pressure	0.049
Temperature + Water Vapour Pressure + Wind Speed	0.049
Atmosphere + Water Vapour Pressure + Humidity + Rainfall	0.049
Atmosphere + Water Vapour Pressure	0.049
Temperature + Water Vapour Pressure	0.049
Water Vapour Pressure + Rainfall	0.049
Temperature + Atmosphere + Water Vapour Pressure + Humidity	0.050
Temperature	0.050
Water Vapour Pressure + Wind Speed	0.050
Atmosphere + Humidity	0.051
Atmosphere + Water Vapour Pressure + Wind Speed + Rainfall	0.051
Wind Speed + Humidity + Rainfall	0.051
Atmosphere + Water Vapour Pressure + Rainfall	0.052
Temperature + Atmosphere	0.052

**Table 3.** Results of six models for monthly incidence of hepatitis E prediction

	RMSE	MAPE (%)	MAE
ARIMA	0.139	48.23	0.130
SVM	0.074	19.46	0.061
GBDT	0.097	23.32	0.078
LSTM	0.060	19.27	0.052
Bi-LSTM	0.054	15.78	0.044
Bi-LSTM + Best Climate	0.044	11.88	0.0377

**Fig. 5.**
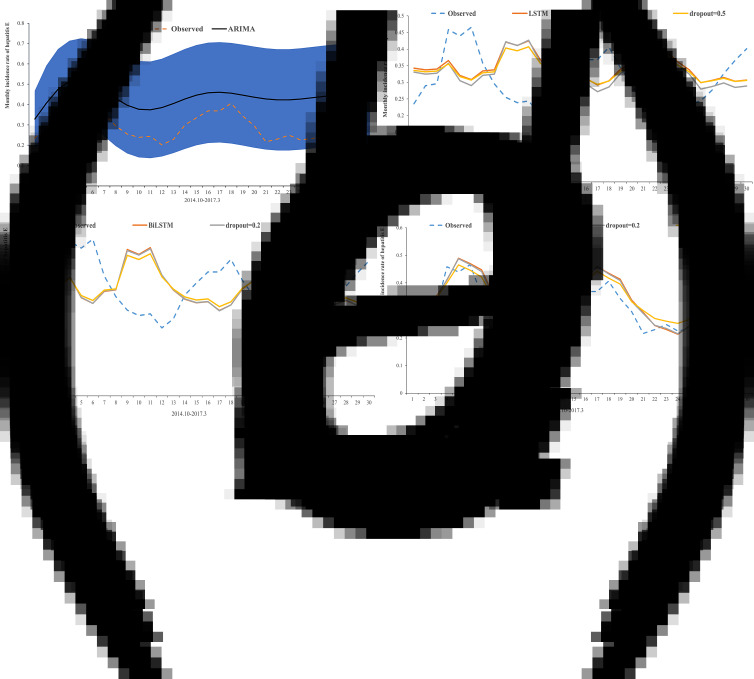
Predictive Intervals of (a) ARIMA model (b) LSTM model (c) BiLSTM model (d) BiLSTM model with best meteorological factors.

Besides, we also compare the predictive intervals of all the models mentioned in Figure 4. For the neural network models, predictive intervals are also possible by adding dropout layers. We show the 95% CI of the ARIMA model and the result of the neural network models with different dropout layers in Figure 5.

## Discussion

Accurately understanding the epidemic trend in advance is essential to the prevention and control of infectious diseases. Hepatitis E is considered an infectious disease mainly confined to areas with poor sanitation and contaminated drinking water supplies. However, as it is also a zoonotic disease and some transmission modes are unknown, more cases have occurred in non-endemic areas including Jiangsu Province, China. Research on its epidemic pattern has drawn extensive attention in recent years, and some researchers have proposed different prediction methods for hepatitis E. For instance, Wang *et al*. [17] use the ARIMA model, Ren *et al*. [16] explore a mixture model using the ARIMA and the back-propagation artificial neural network, and Guo *et al*. [14] adopt the SVM and the LSTM, and Peng *et al*. [15] develop the machine ensemble learning methods, including GBDT and random forest.

This paper attempts to establish prediction models of different types and different complexity by using the monthly incidence rate of hepatitis E from January 2005 to March 2017, including ARIMA, SVM, LSTM, GBDT and Bi-LSTM models (the original Bi-LSTM model and the Bi-LSTM model with meteorological factors). Experimental results show that our Bi-LSTM model with meteorological factors is significantly superior to other models in predicting the monthly incidence of hepatitis E in Jiangsu province. In the prospective prediction stage, its RMSE is less than 0.05, MAPE is less than 20%, and MAE is less than 0.04. The seasonal fluctuation of hepatitis E in the next 30 months is accurately estimated. In this study, when we add the number of layers in the FC layer, the effect does not improve, thus we only used one layer in FC. The decision of batch size depends on the device we use, and with the increase of batch size, the data that the device can compute each time also increases. The number of iterations also depends on the dataset. We find that the model converges at around 220 iterations, so we set iteration to 220. The results also illustrate that when the hyperparameter: timestep is set to 2, the model has the best accuracy. It is consistent with the average incubation period of hepatitis E being near one month [18].

A large number of studies have shown that infectious diseases are sensitive to climate [19–21]. The climate factors may affect the survival and transmission of infectious disease pathogens in the environment, the host susceptibility and exposure opportunities. In recent years, the influence of meteorological factors such as humidity, temperature and rainfall on the epidemic of hepatitis E has attracted extensive attention [14, 16, 22]. However, the model does not show satisfactory performance. In this study, for the establishment of the model, we introduce Bi-LSTM, a model which can capture useful features from both sides. When predicting the incidence rate of a certain month, such a situation is likely to occur that the number of patients in the previous month is small, but the climate conditions of the current month are suitable for the spread of the virus, thus the number of patients increases this month. Based on the characteristics of hepatitis E, we estimate that the number of patients next month will also increase to a certain extent. At this time, we can use the input at the next moment to correct the current prediction. For these common meteorological factors, we compare the influence of their combination and find that the most influential group is using temperature, water vapour pressure and rainfall. As a result, our model has indeed achieved the state-of-the-art performance in predicting the monthly incidence of hepatitis E.

## Conclusion

In this paper, we propose a new Bi-LSTM model with various meteorological factors to predict the monthly incidence of hepatitis E in Jiangsu Province, China, and compared it with existing models using ARIMA, SVM, LSTM and GBDT. The Bi-LSTM model with the meteorological factors of temperature, water vapour pressure, and rainfall can fully extract the linear and non-linear information from the incidence data of hepatitis E and has made significant improvements in interpretability, learning ability, generalisability and prediction accuracy.

## Data Availability

Data supporting the conclusions of this article are included within the article.
